# Development and Novel Therapeutics in Diabetic Retinopathy

**DOI:** 10.2174/0115733998324556240910061514

**Published:** 2025-10-02

**Authors:** Pravinkumar Ingle, Nurin Alesya Hamden, Wai Kei Soh, Rosalina Wibawa Hui Cen Loh, Ketan Hatware

**Affiliations:** 1 Department of Pharmacy Practice, School of Pharmacy, IMU University, Kuala lumpur, Malaysia;; 2 School of Pharmacy, IMU University, Kuala lumpur, Malaysia;; 3 Department of Life Sciences, School of Pharmacy, IMU University, Kuala lumpur, Malaysia

**Keywords:** Challenges in treatment, clinical trials, development, diabetic retinopathy, future therapeutic trends, novel therapeutics

## Abstract

Diabetic retinopathy (DR) is a complication of diabetes mellitus which causes retinal damage which when left untreated will cause visual problems. As the prevalence of DR increases over the years, there is a need to optimise the currently available treatments as well as developing novel drugs to improve the therapy provided for the patients in the clinical practice. Several pharmacological therapies like, anti-vascular endothelial growth factor and anti-inflammatory therapies which include intravitreal, and implant of corticosteroids are significant in the management to decrease the risk of DR-related vision impairment. Clinical trials for novel drug therapies are still ongoing till this day to enhance the efficacy of DR treatment. Even though there are also modern treatments such as laser therapy for the patients, prevention should be done to lower the number of individuals affected by DR. Due to the complexity of DR, there are numerous obstacles to develop new medications for DR which include the increasing healthcare cost of DR treatment. New insights such as utilisation of artificial intelligence will be implemented into the management of DR as it has proved its potential in aiding the screening process. In parallel with the increase in DR prevalence and the number of treatments developed, extensive understanding of the mechanism of action of DR should be further improved to prevent more complications in the future. This review summarises the epidemiological trend, prevention strategies, challenges in treatment, current novel therapeutics (including drugs under clinical trials), future therapeutic trends and possibilities for implementing AI in the early diagnosis and management of DR.

## 1 INTRODUCTION

Diabetic retinopathy (DR) is one of the common long-term complications of diabetes mellitus (DM) that is causing significant visual impairment or blindness which is preventable among the individuals with DM [1, 2]. DR is induced by the breakdown of blood-retinal barrier and the leakage of fluid into the retina which leads to visible lesions such as microaneurysms which can be identified as the earliest manifestation of DR that is visible [3-5].

DR can be categorised into two classifications which are the non-proliferative diabetic retinopathy (NPDR) and proliferative diabetic retinopathy (PDR) [1, 6]. The NPDR is the earlier stage of DR in which vascular permeability and capillary occlusion can be observed in the retinal vasculature [1]. PDR is the advanced stage of DR which can be characterised by the hallmark feature of preretinal neovascularization [6]. Another category is diabetic macular oedema (DME) which is the major factor of blindness in patients with DR [1, 6]. Individuals may develop DME regardless of the severity or stages of DR, though those with more severe DR have higher chances to develop DR.

## 2 METHODS

In this review, we used keywords combinations search of “diabetic retinopathy”, “epidemiology”, “treatment strategies”, “future therapeutic trends”, “development”, “novel therapeutics”, “clinical trials”, “challenges”, and “prevention” in Google and PubMed to review novel treatment strategies and future directions for DR treatment.

We categorized treatment and prevention strategies for DR in below groups: (1) epidemiology; (2) novel treatments strategies in DR; (3) drugs under clinical trials; and (4) prevention and challenges in treating DR. Based on this discussion, we also provided the future directions and conclusion.

## 3 EPIDEMIOLOGY

The occurrence of DR has rapidly grown along with the increase of diabetic patients worldwide. There were 285 million of adults in the range of 20 to 79 years old who were affected by diabetes mellitus (DM) in 2010 as estimated by the International Diabetes Federation [7]. It was predicted that the number will keep increasing drastically to 552 million in 2030, 642 million by 2040 and 700 million in 2045 [6-8]. In 2010, it was revealed that among 32.4 million people with blindness, 0.8 million of people were visually impaired due to diabetic retinopathy [3]. The number increased rapidly in which it was revealed that DR contributed to 1.1% of 36 million people with visual impairment in 2015 [6]. In 2020, the prevalence rate of DR reached 34.6% globally and even higher in developed countries in which the rate was close to 40.3% which affected 3.6% of type 1 DM patients and 1.6% of type 2 DM patients [9]. The prevalence is expected to rise through 2045 affecting Middle East countries, North Africa and the Western Pacific [6].

However, there has been a declining trend of the number of individuals affected by DR in developed nations, especially the United States. This declining trend may be contributed by the increasing number of screening services for DR and improvements in systemic controls [4, 7]. In spite of that, cases of visual impairment due to DR have surged drastically on a global scale.

DR is significantly more prevalent in individuals with type 1 DM compared to type II DM [8]. Individuals who have had type I DM for more than 20 years have higher chances of developing DR in comparison to individuals who have had type II DM for 10 years [8]. Duration of diabetes is a significant risk factor which is non-modifiable for the development of DR. Some of the modifiable risk factors of developing DR include glycemic control and higher systolic blood pressure [10]. It is proven that good control of HbA1c leads to lower risk of DR [11].

The prevalence of DR is increasing day by day and researchers have been focusing on this emerging problem more significantly in the last few decades. This can be observed on the official NCBI website and is summarised in Fig. (**1**).

## 4 PATHOGENESIS

Among working-age adults, DR is a common complication of diabetes mellitus and the leading cause of vision impairment and blindness. DR is the result of a complex interaction between inflammatory, vascular, and metabolic processes. Here are the possible main mechanisms outlined. Persistent hyperglycemia is the main cause of DR pathogenesis. The blood vessels in the retina are damaged by elevated blood glucose levels. Advanced Glycation End Products (AGEs) hyperglycemia leads to the formation of AGEs, which accumulate in retinal tissues and causes inflammation and vascular damage. There are several mechanisms involved in pathogenesis of DR including microvascular changes, reactive oxygen species, inflammatory biomarkers release (*e.g.*, TNF-α, IL-1β, and IL-6), diabetic macular edema, VEGF over expression, activation of renin angiotensin system activation led to ocular vasoconstriction, ischemia and neovascularization [12-22].

The development of DR can be prevented or slowed down by tight glycemic control, early diagnosis through routine eye examinations, and other lifestyle related measures. [23] To DR in its later stages, treatments that target VEGF, inflammation, and oxidative stress are essential. Additionally, the use of AI-related tools could help patients with early diagnosis and management of DR.

## 5 CONVENTIONAL AND NOVEL TREATMENTS STRATEGIES IN DR

### 5.1 Anti-VEGF

In DR, vascular endothelial growth factor (VEGF) has a significant role in the development of macular oedema (ME) and the proliferation of new vessels that will result in vitreous haemorrhage [24, 25] Intravitreal anti-VEGF injections, which are the mainstay of exudative macular degeneration treatment, have been demonstrated to be highly effective in diabetic macular oedema [25]. Despite being the first anti-VEGF to receive approval, pegaptanib sodium, a selective pegylated ribonucleic acid aptamer that blocks VEGF165, was promptly superseded by more potent drugs of the same class that blocks all isoforms of VEGF [25, 26]. In 2015, ranibizumab 0.3 mg/0.05 mL was approved by FDA for the treatment of NPDR and PDR with DME [10, 27]. In addition, the indication of aflibercept 2.0 mg /0.05 mL was expanded by FDA in 2019 for the treatment of all stages of DR, both with and without DME. [28, 29] Besides, bevacizumab 1.25 mg/0.05 mL is presently used off-label to treat a variety of neovascular ophthalmic diseases [29].

The data from DRCR Protocol T demonstrated that the efficacy of ranibizumab, aflibercept, and bevacizumab for DME was comparable when the initial loss of visual acuity (VA) was minor (baseline vision better than 20/50) [30]. However, individuals who had baseline vision of 20/50 or worse responded better to aflibercept than other anti-VEGF agents [30, 31]. Among the three agents, bevacizumab thinned the retina less, but the visual acuities remained stable for up to two years. All three anti-VEGF groups showed improvement in visual acuity at two years, with fewer injections [31].

The latest drug, brolucizumab, the smallest molecular weight of an anti-VEGF agent, was approved by FDA for a 12-week dosage schedule after three monthly loading doses [26, 32]. Noteworthy, brolucizumab is currently the only anti-VEGF agent that extends beyond 8-week intervals and has the least number of injections per year [26]. Thus, it provides the greatest benefit from a societal perspective as it is substantially cost-saving compared to other anti-VEGF agents. However, subconjunctival haemorrhage and reduced visual acuity are the most common side effects of intravitreal brolucizumab. Meanwhile, iritis and uveitis were the most prevalent inflammatory side effects [33].

Faricimab, a humanised bispecific immunoglobulin G1 (IgG1) antibody that acts as both VEGF-A and ANG2 signal inhibitors [34]. Rush *et al.*'s study of treatment-resistant DME showed that the mean of best-corrected visual acuity was significantly improved after switching therapy from intravitreal aflibercept injection to intravitreal faricimab in 4 months [34, 35]. As a result, faricimab may be an optimal treatment option for subjects recalcitrant to aflibercept therapy for fewer intravitreal injections without compromising visual outcomes.

Anti-VEGF (vascular endothelial growth factor) drugs are used in the treatment of conditions involving excessive blood vessel growth, such as age-related macular degeneration (AMD) and certain types of cancers. These drugs work by inhibiting the release of excess VEGF, a protein that promotes the formation of new blood vessels. In circumstances like hypoxia, cells secrete VEGF. A signaling cascade is initiated when VEGF binds to endothelial cell surface VEGF receptors (VEGFR). The tyrosine kinase activity in the receptor is activated by VEGF binding to its receptors. This activation triggers a sequence of intracellular signaling processes that promote the migration, proliferation, and angiogenesis of endothelial cells. Anti-VEGF medications work by binding to VEGF and preventing it from interacting with endothelial cell receptors. Additionally, the signaling pathways necessary for the formation of new blood vessels are inhibited. This delays the formation of new, abnormal blood vessels, which can help slow the progression of conditions such as AMD and certain types of cancer. Some common Anti-VEGF drugs include, bevacizumab, ranibizumab, aflibercept, *etc.* [3, 4, 8, 10].

Possible mechanisms of action of common anti-VEGF drugs in the management of DR are outlined in Fig. (**2**).

### 5.2 Anti-inflammatory Therapy

#### 5.2.1 Intravitreal Corticosteroids

Corticosteroids are potent anti-inflammatory and anti-oedematous drugs that inhibit the production of proinflammatory mediators involved in DME and decrease VEGF synthesis [36]. Besides, corticosteroids also suppress the production of thromboxane, leukotrienes, and prostaglandins, which improves retinal oxygenation and the blood-retinal barrier (BRB) [36, 37]. Therapy with corticosteroids is usually reserved for anti-VEGF non-responders or refractory DME individuals.

Based on DRCR Protocol I trial, off-label intravitreal triamcinolone was demonstrated to be as effective as anti-VEGF in decreasing macular thickness and increasing VA in patients with DME through a follow-up period of 24 weeks [38]. However, risks of increased intraocular pressure (IOP) and cataracts are associated with the long terms use of intravitreal triamcinolone [39]. During the two-year follow-up, 50% of patients showed an increased in IOP, and 59% of patients in the triamcinolone group received cataract surgery [1]. However, a study reported that intravitreal injection of preserved triamcinolone showed a higher occurrence of IOP than preservative-free triamcinolone [40].

#### 5.2.2 Corticosteroids Implant

The biodegradable dexamethasone (DEX) intravitreal implants deliver DEX into the vitreous cavity through a sustained-release method [36]. The effectiveness of dexamethasone delivery system's assures prolonged medication exposure and significantly lowers the need for injections. The average number of injections for three years in the DEX implant study (0.7 mg and 0.35 mg) in patients with DME was four. Compared to patients in the sham group, a greater proportion of patients who received the DEX implant experienced a statistically significant reduction in central macular thickness [41]. Cataract development and IOP elevation are the major side effects of DEX implants. Besides, visual improvement associated with DEX implant therapy was substantial in phakic eyes until cataracts developed, and improvement in baseline vision was regained after cataract surgery [36, 41].

The newer generation of non-bio erodible fluocinolone acetonide implants (Iluvien) delivers the drug as a sustained-release method for up to 36 months. A study demonstrated that the percentage of patients who received Iluvien-FA implants (0.2 μg/day or 0.5 μg/day) gained BCVA improvement from a baseline of ≥15 letters were significantly higher compared to patients in the sham group at 36 months [42]. In 2014, Iluvien-FA implants (0.19 mg) were approved by the FDA to treat chronic DME or patients who are insufficiently responsive to existing treatment without a clinically significant increase in IOP [42, 43].

### 5.3 Drugs Under Clinical Trials

Clinical trials are being conducted on several drugs to treat diabetic retinopathy. These medications target various pathogenesis-related biomarkers and mechanisms that are involved in diabetic retinopathy, including VEGF, NO signaling, oxidative stress, and ocular inflammation. They also prevent BRB breakdown, neuroinflammation, angiogenesis, and cell death.

These drugs encompass a wide range of treatment approaches aimed at controlling and potentially reversing the progression of diabetic retinopathy. They are currently in various phases of clinical trials, and their success hinges on the results of these studies.

#### 5.3.1 Non-steroid Anti-inflammatory Drugs

IL-6 is assumed to be one of the main proinflammatory cytokines involved in DME development as vitreous IL-6 humour levels were consistently found to be elevated in patients with DME [44]. Currently, new IL/IL-6R inhibitors are being assessed in preclinical research and clinical trials. A trial conducted on the humanised monoclonal antibody, EB-031(NCT02842541) that potentially binds to IL-6 and inhibits IL-6 intraocular signalling activity [45]. Besides, READ-4 study compared the results of effectiveness and safety of monotherapy humanised monoclonal IL-6 antibody tocilizumab (NCT02511067) and in combination with ranibizumab in eyes with DME which have not been published yet [46].

#### 5.3.2 Other Therapeutic Agents Under Clinical Trials

##### 5.3.2.1 Runcaciguat (BAY 1101042) (NCT04722991)

Novel approach targeting early microangiopathic damage might result in more effective DR treatment outcome. NO (nitric oxide)/ cyclic GMP modulator plays an important role in NO signalling which involve in DR progression [47]. Runcaciguat, the soluble guanylate activator which showed anti-inflammatory effect in DR animal models, is undergoing clinical trials to investigate its safety and efficacy in treatment of DR [48].

##### 5.3.2.2 Darapladib (NCT01506895)

Lipoprotein-associated phospholipase A2 (Lp-PLA2) is essential for development of atherosclerosis and vascular leakage in animal models. In diabetic rats, darapladib which is a Lp-PLA2 inhibitor stabilize BRB by significantly suppressing BRB breakdown [49]. Thus, Lp-PLA2 could be a potential therapeutic target for treatment of DME. In a Phase IIa study, darapladib showed substantial improvement in BCVA as compared to the placebo group [50].

##### 5.3.2.3 RG7774 (NCT04265261)

Cannabinoid receptors, CB2 are expressed in the brain and retina. It has been demonstrated that CB2 receptors activation reduces inflammation, oxidative damage, and cell death [51]. Therefore, utilization of CB2 receptors as an anti-inflammatory will enhance a prospective approach to the treatment of DR. Currently, the safety and effectiveness of oral administration CB2 receptor agonist, RG7774 is being assessed under phase II clinical trial [52].

##### 5.3.2.4 Luminate (ALG-1001) NCT02348918

Integrins are essential for cell signalling and regulation, and the proliferation of new blood vessels. Luminate which acts as an integrin receptor antagonist promotes vitreolysis and prevents angiogenesis through the inhibition of integrin receptors [53]. A clinical trial compared the safety and effectiveness of monotherapy luminate, alone and combination therapy with bevacizumab [54]. The study revealed a slight improvement in mean BCVA in two groups of patients treated with luminate with bevacizumab compared with bevacizumab alone [53, 54].

The details of the novel and potential drugs currently under clinical trials are provided in Table **1**.

### 5.4 Surgical Interventions Including their Indications and Contraindications in DR

When severe complications from DR arise and cannot be effectively treated with medication or laser therapy, surgical options are typically considered. Additionally, every DR case requires a personalised approach, and the decision to proceed with surgery should be discussed with a diabetic retinal specialist, considering the patient's overall health, the severity of the retinal disease, and the potential for visual improvement. The following outlines the possible reasons for surgical intervention and contraindications.

Nearly all medical specialties use laser therapy extensively as it is effective in treating retinal vascular diseases. In addition to being used in the operating room as part of the surgical treating ailments such as tractional retinal detachments in DR, retinal laser therapy is also applied to treat same conditions in ambulatory clinic settings [62, 63]. Retinal photocoagulation is being broadly employed since 1960s and the development of pan retinal photocoagulation (PRP) has revolutionised the therapy of PDR as it immediately evolved as the gold standard following the accomplishment of DR Study and the Early Treatment Diabetic Retinopathy Study (ETDRS) [64-66]. From the studies, PRP was proven to be helpful in lowering the possibility of extreme vision loss when compared to eyes that were not treated in PDR and NPDR. These findings verified PRP as the foundation of care for PDR and life-threatening NPDR patients and it is still currently the primary treatment [64, 66]. However, over the past decade, intravitreal anti-VEGF medicines have produced positive findings for the treatment of PDR with more functional results compared to PRP [67, 68]. It is thought to have promising effects on neovascular (NV) regression in patients with active PDR despite its short duration. Numerous studies showed that PRP combined with anti-VEGF medications had superior short-term anatomical functions in high-risk PDR patients. Meanwhile, other studies shown that neither in placebo group nor patients with persistent NV following PRP, could achieve full regression of NV fully with or without anti-VEGF medications [69].

Vitrectomy is a surgical procedure which allows access to both vitreous and retina [70, 71]. It indicates in DR for few conditions including non-resolving vitreous haemorrhage, dense sub hyaloid haemorrhage, tractional retinal detachment, combined retinal detachment, vitreomacular traction and taut posterior hyaloid causing vitreopapillary traction [72, 73]. Vitrectomy insists to remove the vitreous gel during the surgery and thus resulting a rapid clearance of haemorrhage, eliminating traction on neovascular vessels that may cause recurrent vitreous haemorrhage and lastly treating neovascularization through intraoperative delivery of photocoagulation [70, 74]. PDR had been one of the causes of vitreous haemorrhage and the ultimate aim of the treatment is to minimise the risk of acute or severe vision loss. Since the 1970s, vitrectomy had become the ordinary therapy for non-clearing vitreous haemorrhage. Despite the surgical techniques had shown improvement lately, there is still a possibility for occurrence of surgical complications. Therefore, non-surgical approaches would still be the mainstay approach [70].

Macular laser therapy, known as threshold laser therapy, uses continuous wave laser to generate a visible burn in the retina. Despite its mechanism is still unclear, it is said to give effect on retinal pigment epithelium cells and this therapy can also be undertaken using subthreshold micropulse laser [75]. Micropulse transscleral cyclophotocoagulation (MP-TSCPC) is another technique that enables a series of quick, repeating spikes of energy following by extended rest periods. Through this method, heat produced by laser conduction can be drained during nonoperational cycle. Therefore, localized coagulative effects in the ciliary body can be induced by the energy given in micropulse mode meanwhile minimizing overheating and thermal injury to the surrounding tissues [76].

### 5.5 Indications for Surgical Interventions

Vitrectomy is used for patients with vitreous hemorrhage that does not clear on its own, tractional retinal detachment, and severe PDR, diffuse diabetic macular edema that is unresponsive to medical or laser therapy, pre-macular fibrosis that may cause substantial vision loss or alteration. Endolaser photocoagulation is used to treat active neovascularisation during vitrectomy, reducing the risk of recurrent bleeding and further neovascularisation. Focal/Grid laser treatment is used to treat macular edema, which can occur in both non-proliferative and proliferative stages of diabetic retinopathy. Endolaser treatment is an intraocular laser treatment performed during a vitrectomy to treat proliferative changes and stabilise the retina in diabetic retinopathy. Scleral buckling is mainly used in cases of retinal detachment, including those caused by diabetic retinopathy. It is a surgical procedure in which a silicone band is placed around the eye to push the wall of the eye against the detached retina [24, 27, 28].

### 5.6 Contraindications for Surgical Interventions

Diabetic specialist surgeons who treat advanced stages of DR often perform various types of surgeries. However, before undergoing this type of surgery, there are several contraindications to consider. Patients with uncontrolled diabetes and poor glycemic control may be at an increased risk for surgical complications and delayed healing. Patients with active infections, such as ocular or periocular infections may be at higher risk for postoperative endophthalmitis. Patients with severe cardiovascular or other systemic diseases that make general anesthesia or prolonged surgery risky should be carefully evaluated before proceeding with any surgical intervention for DR. Patients with poor visual potential or no light perception vision are unlikely to benefit from surgical intervention to improve visual outcomes. Patients with severe neovascular glaucoma, in which intraocular pressure is not controlled despite using drug therapy, are at higher risk for surgical complications and less favorable outcomes [29, 30, 62].

A summary of both conventional and novel approaches for managing DR is provided in Fig. (**3**).

## 6 PREVENTION OF DR

DR is one of the most common leading causes of blindness worldwide. According to a previous study, 10% of Type-2 DM patients was revealed to experience retinopathy that poses a risk to the eyesight and might result in blindness [66, 77]. Therefore, it is crucial to manage the systemic conditions in diabetic patients effectively to prevent vision loss [78]. The prevention strategies to combat the global blindness due to DR had been designated into primary, secondary and tertiary prevention [60]. Primary prevention is targeting to restrain or detain the onset of DR [77, 79]. One of the most critical steps is to undergo a DR screening for early detection. Pharmacological interventions are often applied for improvement on blood sugar and blood pressure level [79]. Lowering A1C along with a good blood pressure control with systolic and diastolic pressures of lesser than 130 and 80 mm Hg respectively will diminish the rate and progression of DR [78]. Additionally, effective diabetic care could be achieved by self-management [72, 74]. For illustration, individuals with diabetes but without symptoms of DR should adjust own lifestyle, behaviour and improve self-awareness of DR [79]. Regular physical exercise contributes to decrease the incidence of DR [78]. It has been revealed that adoption of healthy behaviours among T2DM patients has greatly delayed or prevented the onset of DR [77].

Secondary prevention aims to prevent the progression of DR to vision-threatening stages in patients with early stages of DR. DR screening and monitoring are required for detecting the progression of earlier stages of DR to vision-threatening DR. Similar to primary prevention, pharmacological treatment is applied to control glycaemic and blood pressure level. DR management in different countries will be based on the enforcement of guidelines and policy to each their own health services system. Tertiary prevention involves laser therapy, anti-VEGF therapy and vitrectomy as its ultimate goal are to avoid lacking vision, rehabilitate vision and boost quality of life [79-82].

## 7 CHALLENGES IN TREATING DR FROM DIFFERENT PERSPECTIVES

DR is a complex ocular disorder due to its multifactorial nature. The key challenge in treatment of DR is the exploration of drugs that can target numerous pathways involved in DR development [3, 83]. Adverse effects from the drugs demand significant financial resources that may result in loss of working days and decreased quality of life [30]. As the prevalence of DR is going to rise over the coming years, the health care costs of DR are expected to increase fourfold from 2017 to 2025. The whole expenditure is split between the Health Systems and the patients themselves who need to attend for recurrent appointments and treatments [83]. Moreover, the access to health care services is often inadequate and difficult hence patients in developing countries might decide to remain untreated or unable to be treated completely [84, 85]. On the other hand, one of the most challenging parts in governing DR is due to the undiagnosed diabetes as DR may evolve before a diagnosis of diabetes thus patients might experience symptoms of diabetic complication [84]. With the purpose of early detection of DR to prevent higher prevalence in blindness, regular screening is being recommended. However, the increasing numbers from 415 million in 2015 to an estimated 642 million in 2040 had marked a substantial challenge in screening process [85]. Additional difficulty arises from the lack of well-trained professionals to provide effective screening and DR treatment [86]. The healthcare providers also concern on the poor awareness of DR among the patients and find it difficult to explain the diseases due to language barrier. Patients themselves who lack motivation, social support and improvement in vision tend to stop the treatment which eventually contribute to higher risk of developing DR [87-90].

## 8 FUTURE DIRECTIONS AND MANAGEMENT OF DIABETIC RETINOPATHY WITH ARTIFICIAL INTELLIGENCE (AI)

There have been many clinical developments of therapeutic agents such as oral fenofibrate which have shown promising results in clinical trials to slow down the progression of DR [91]. Oral fenofibrate could be considered as a treatment option not only for adults, but for the paediatric patients once the effects of long-term use have been fully investigated [40]. According to the Fenofibrate Intervention and Event Lowering in Diabetes (FIELD) study, a noticeable reduction of relative risk needing the laser treatment can be observed in those administered with fenofibrate [91]. Since almost all the treatments available for DR are considered invasive, topical treatments such as Alpha-2-agonists like brimonidine (brimonidine 0.1%) alone or combined with somatostatin (brimonidine 0.2% + somatostatin 0.1%) are still under investigation in which may be beneficial for paediatric patients [92]. Developing new potential biomarkers is crucial as it facilitates a better understanding of DR and contributes into exploring novel therapy options to prevent vision impairment in people with diabetes. As there are available and existing therapeutic interventions of DR which are focused on the late stage of the disease, the key for future study is to develop novel biomarkers which should be accessible, non-invasive, inexpensive and accurate in assessing the early stages and progression of the disease1 [93]. There are several potential systemic and ocular biomarkers that may serve to evaluate the presence and progression of DR such as TNF-α, TGF-β, IL-6 and IL-8. Increased levels of circulating TGF-β is observed to be strongly related to the incidence of DR which TGF-β1, in particular, had sensitivity of 72% and specificity of 88% to predict the presence of DR. In addition, diabetes has been linked with the increased levels of the pro-inflammatory molecules TNF-α and interleukins (Ils) in the blood. It has been observed that high levels of IL-6 and IL-8 in the body have been linked to the presence of DR and correlated to the severity of the disease [94]. As pro-inflammatory and angiogenic molecules have been used in basic science studies up to now, it is highly anticipated that at least some of the biomarkers will be utilized in clinical practice in the future. As early detection of DR is the key to a better treatment, extensive research should be carried out to identify novel biomarkers which are useful and beneficial for DR patients to reduce the risk of vision loss. Development of new therapies which are more effective and better-tolerated will be accompanied by increasing further knowledge of the progression and development of DR.

Implementation of AI into the treatment of DR has gained a lot of interest as it has been utilised in the diagnosis of conditions related to the eye and the potential has not been fully utilised yet [95]. Applying AI techniques into modern healthcare may improve the treatment of ocular disorder while reducing the healthcare cost. It has been revealed that AI can analyse details on simple fundus photos which are undetectable by humans. Hence, implementing AI will provide higher accuracy during the screening of DR which will indirectly reduce the needs of skilled manpower to do image grading manually [96].

Technologies for retinal imaging have been adapted into clinical practice which has been useful in order to document the development of DR over time. The databases have contributed to the development of technologies such as deep learning algorithms, mathematical models and AI which are crucial in diagnosing, grading severity, assessing risk of progression and predicting response to treatment [10]. The highly promising performance of AI in the screening of DR has been increasing the chances of it being fully implemented into the future treatment plan even though there are still some challenges that may limit the usage of AI in clinical practices [97]. The usage of AI-based techniques in the treatment or diagnosis of DM needs further investigation as it is an alternative that is cost-effective to reduce complications such as blindness or visual loss [98].

Currently, the treatment and management of diabetic retinopathy has shown considerable potential with AI. The following are some ways in which AI is being used and implemented to help diagnose and manage DR and its complications.

Early Diagnosis: AI can efficiently and accurately screen big data to identify patients who may need additional testing by diabetic retinopathy experts. AI models can detect early changes in the retina that may be missed by a human examiner, leading to earlier and more accurate diagnoses. In their early stages, these systems can often detect microaneurysms, hemorrhages, and other abnormalities of the retina. In primary care settings and remote locations, AI-driven solutions can be used to enhance screening accessibility for patients who may not have convenient access to specialised eye care. Remote screening can be made easier with AI-driven diagnostic technologies, which allow primary care physicians to capture retinal images and submit them to specialised AI systems for evaluation. This is particularly beneficial in isolated or remote areas [13, 99-103].

Monitoring disease progression with predictive analytics: AI can help monitor the progression or regression of diabetic retinopathy by comparing successive retinal images. This also helps identify patients who are at high risk and need more frequent monitoring. Using imaging data and patient information to predict the likelihood of developing an illness and tailor personalized treatment plans [102-104].

Treatment optimisation: AI is also being used to improve diabetic retinopathy treatment techniques. AI can assist in identifying the most efficient treatment plans, including the frequency and timing of interventions such as anti-VEGF injections, laser therapy, or combination therapy. AI-driven data analysis can also expedite the discovery of novel therapeutic medications by locating possible targets and forecasting the effectiveness of the medication [105-108].

Patient education and support: AI-powered chatbots and smartphone apps can inform patients about diabetic retinopathy, schedule appointments for them, and provide advice on how to care for their disease. AI can monitor a patient's lifestyle choices and offer tailored advice to help them better control their diabetes, thereby reducing the risk of retinopathy. Retinal images can be taken at home by patients using devices, and experts can remotely examine them [98, 102-109].

Robotic surgery innovation: Robotic technologies driven by AI can assist surgeons in performing delicate surgeries with greater precision. This is crucial because, in retinal procedures, precision is vital to avoid damaging the delicate eye tissues. Laser technology advancements, such as pattern scanning laser photocoagulation, offer more accurate and less harmful treatment options [106, 110].

We have also included some examples of AI-related systems currently in use. The FDA has recently approved the marketing of an artificial intelligence-based device, called IDx-DR, to detect certain diabetes-related eye problems. It is the first device approved for commercialization that makes a screening decision without requiring a clinician to additionally interpret the image or data, health care professionals who might not typically be involved in eye care can use it [109, 110].

Through the analysis of retinal images, Google's DeepMind has developed an artificial intelligence system that can accurately identify over 50 eye disorders, including diabetic retinopathy. Diagnosis times could be sped up by using this device, which analyzes 3D retinal scans [111].

By improving early diagnosis, tracking the progression of the illness, maximising appropriate treatment selection, and enhancing patient management, artificial intelligence is revolutionising the field of diabetic retinopathy treatment. The implementation of AI tools will be more effective in early diagnosis, prevention, and management of DR and its related complications.

## CONCLUSION

In conclusion, DR remains the major cause of impaired vision among patients with DM. Further understanding of the disease is needed to improve the development of therapeutic agents for DR. Current available treatments should be optimized to enhance the quality of life for patients. It is crucial to better understand the risk factors and mechanisms of DM to develop more treatment options to reduce ophthalmic complications. Additionally, in order to fully understand AI's potential in treating this prevalent condition, more research and collaboration between AI experts and healthcare providers are necessary.

## STUDY LIMITATIONS

The limitation of this study may lie in its methodological approach, which relies more on specific keywords and secondary data than primary evidence. Recent therapeutic advancements and ongoing clinical studies have not been included in the analysis. Additionally, inconsistent research designs and a lack of long-term practical results could restrict the applicability of the findings to various patient populations.

## Figures and Tables

**Fig. (1):**
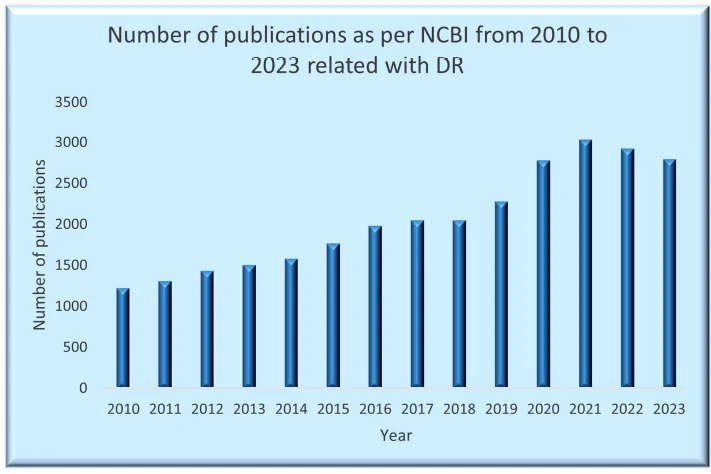
The focus of researchers as evidenced by NCBI publications from 2010 to 2023, indicates an increased prevalence of DR.

**Fig. (2):**
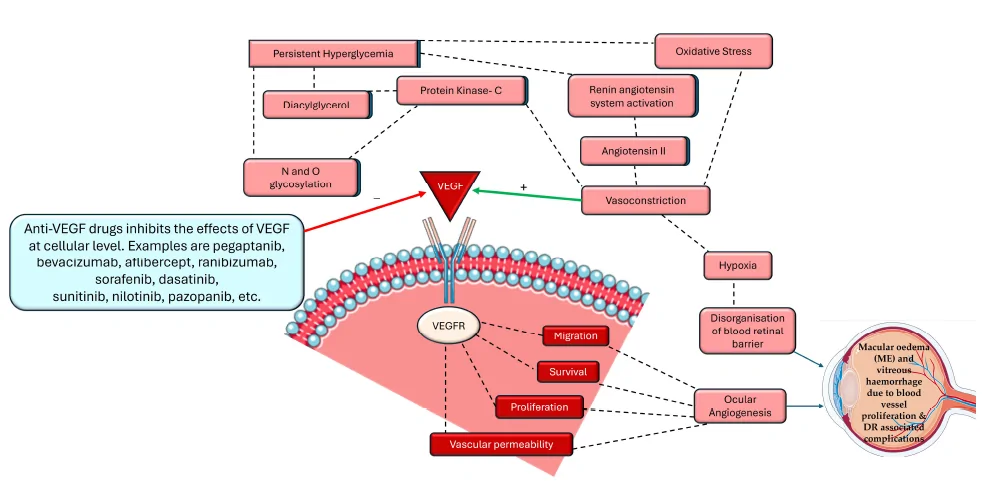
Possible mechanism of action of common anti-VEGF drugs in the management of DR.

**Fig. (3):**
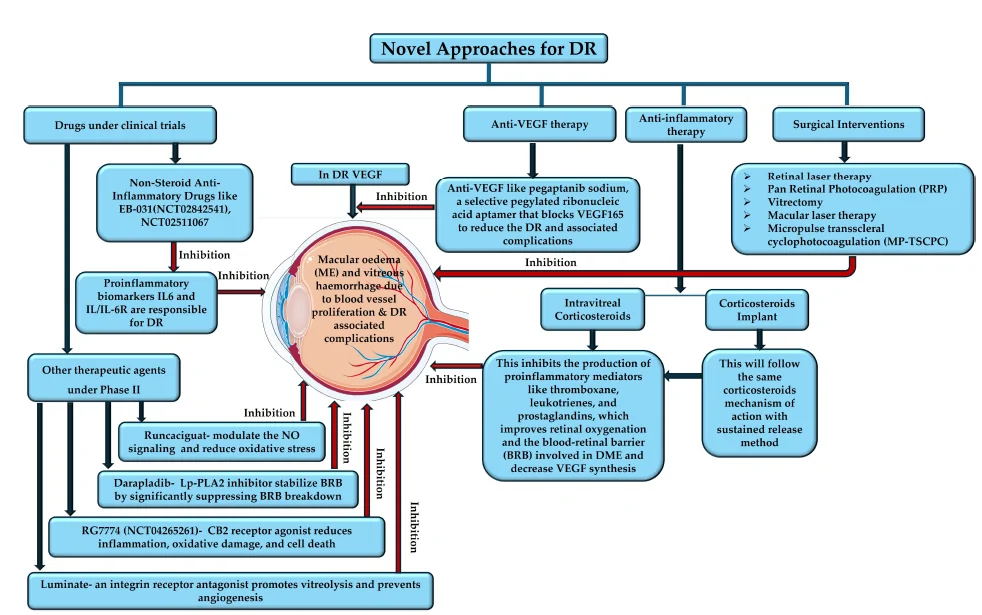
Conventional and novel approaches for the management of DR.

**Table 1:** Novel drugs under clinical trials.

** Sr. No. **	**Drug Name**	** Possible Mechanism **	** Status **
1	Runcaciguat	Soluble guanylate cyclase (sGC) stimulator targets the nitric oxide signaling pathway.	Phase II [48]
2	AKB-9778	Inhibition of the protein tyrosine phosphatase PTP1B and it also involves restoration of Tie2 signaling.	Phase II [55]
3	BI 764524	Chemokine (C-C motif) ligand 2 (CCL2) inhibitor.	Phase II [56]
4	RGX-314	VEGF inhibition through gene therapy delivering a gene encoding.	Phase II [57]
5	Nesvategrast (OTT166)	It is a selective RGD integrin inhibitor.	Phase II [58]
6	Tarcocimab	Anti-VEGF therapy.	Phase III [59]
7	APX3330	It inhibits Ref-1 (reduction oxidation effector factor-1 protein), which controls factors related in ocular angiogenesis and inflammation.	Phase II [60]
8	EXN407	It is having pro-angiogenic activity through inhibition of SRPK1 selectively.	Phase II [61]

## LIST OF ABBREVIATIONS

DR: Diabetic Retinopathy
DM: Diabetes Mellitus
NPDR: Non-proliferative Diabetic Retinopathy
PDR: Proliferative Diabetic Retinopathy
DME: Diabetic Macular Oedema
